# The Ability of the Nottingham Hip Fracture Score to Predict Mobility, Length of Stay and Mortality in Hospital, and Discharge Destination in Patients Admitted with a Hip Fracture

**DOI:** 10.1007/s00223-020-00722-2

**Published:** 2020-07-11

**Authors:** Radcliffe Lisk, Keefai Yeong, David Fluck, Christopher H. Fry, Thang S. Han

**Affiliations:** 1 Department of Orthogeriatrics, Ashford and St Peter’s NHS Foundation Trust, Guildford Road, Chertsey, Surrey KT16 0PZ UK; 2 Department of Cardiology, Ashford and St Peter’s NHS Foundation Trust, Guildford Road, Chertsey, Surrey KT16 0PZ UK; 3 School of Physiology, Pharmacology and Neuroscience, University of Bristol, Bristol, BS8 1TD UK; 4 Institute of Cardiovascular Research, Royal Holloway, University of London, Egham, Surrey TW20 0EX UK

**Keywords:** Geriatrics, Health economics, Two-graph ROC analysis

## Abstract

The Nottingham Hip Fracture Score (NHFS) has been developed for predicting 30-day and 1-year mortality after hip fracture. We hypothesise that NHFS may also predict other adverse events. Data from 666 patients (190 men, 476 women), aged 60.2–103.4 years, admitted with a hip fracture to a single centre from 1/10/2015 and 7/12/2017 were analysed. The ability of NHFS to predict mobility within 1 day after surgery, length of stay (LOS) find mortality, and discharge destination was evaluated by receiver operating characteristic curves and two-graph plots. The area under the curve (95% confidence interval [CI]) for predicting mortality was 67.4% (58.4–76.4%), prolonged LOS was 59.0% (54.0–64.0%), discharge to residential/nursing care was 62.3% (54.0–71.5%), and any two of failure to mobilise, prolonged LOS or discharge to residential/nursing care was 64.8% (59.0–70.6%). NHFS thresholds at 4 and 7 corresponding to the lower and upper limits of intermediate range where sensitivity and specificity equal 90% were identified for mortality and prolonged LOS, and 4 and 6 for discharge to residential/nursing care, which were used to create three risk categories. Compared with the low risk group (NHFS = 0–4), the high risk group (NHFS = 7–10 or 6–10) had increased risk of in-patient mortality: rates = 2.0% versus 7.1%, OR (95% CI) = 3.8 (1.5–9.9), failure to mobilise within 1 day of surgery: rates = 18.9% versus 28.3%, OR = 1.7 (1.0–2.8), prolonged LOS (> 17 days): rates = 20.3% versus 33.9%, OR = 2.2 (1.3–3.3), discharge to residential/nursing care: rates = 4.5% vs 12.3%, OR = 3.0 (1.4–6.4), and any two of failure to mobilise, prolonged LOS or discharge to residential/nursing care: rates = 10.5% versus 28.6%, 3.4 (95% CI 1.9–6.0), and stayed 4.1 days (1.5–6.7 days) longer in hospital. High NHFS associates with increased risk of mortality, prolonged LOS and discharge to residential/nursing care, lending further support for its use to identify adverse events.

## Introduction

Hip fracture, a life-changing event, is associated with prolonged hospitalisation, disability and mortality, as well as discharge to residence with higher levels of care [1–4]. These adverse outcomes impose enormous personal and social costs [5], which continues to escalate given the increasing numbers of older individuals living in most western societies, including the UK [6]. Information on those who are at greatest risk of adverse outcomes after a hip fracture is crucial both to patients and healthcare teams to allow for appropriate arrangements of discharge planning—either a return to the patient’s own home, a temporary period of rehabilitation, or a permanent change of residence to a higher level of care such as a residential or nursing home [7, 8]. The Nottingham Hip Fracture Score (NHFS) was originally developed to predict 30-day mortality [9], and subsequently 1-year mortality after a hip fracture [10], therefore the majority of studies have been restricted to analysis of post-discharge mortality. However, there is also evidence that NHFS is inversely associated with the proportion of patients discharged back to their own homes [11].

The NHFS contains a number of components that reflect poor health including older age, low haemoglobin levels, cognitive impairment, co-morbidities and institutional residence. Therefore, we hypothesise that NHFS may also predict other adverse events commonly observed in patients admitted to hospital with a hip fracture. In this study, we sought to examine the ability of the NHFS to predict other adverse events: mobility within 1 day after surgery for a hip fracture (an indication of rapid functional recovery); length of stay (LOS) and mortality in hospital; and discharge destination for patients admitted with hip fractures. Furthermore, we aimed to derive NHFS thresholds to identify individuals at increased risk of these adverse outcomes.

## Methods

### Study Design, Participants and Setting

We conducted a cross-sectional study of older individuals admitted with hip fractures to a National Health Service hospital, serving a catchment population of over 410,000 people.

### Measurement

Through our participation in the National Hip Fracture Database (NHFD) Audit Programme [4, 12, 13], data were prospectively collected by a Trauma Coordinator for patients admitted with a hip fracture from time of admission to discharge. The data comprised clinical characteristics and care quality, mobility within 1 day after hip surgery, LOS during admission, and discharge destination. Pre-existing co-morbidities were identified from electronic record databases by disease codes categorised by the International Classification of Diseases [14]. All data were updated regularly into a database managed by the lead orthogeriatrician who checked the data to ensure completeness and accuracy. Further data inspection was carried out independently by an independent investigator. Any anomalous data were resolved by these two authors by examining the original electronically stored data recorded by our hospital. There were only a few minor errors that required rectification. Demographic and clinical information was detailed including: age; sex; residency prior to admission; dates of admission; surgery; death or discharge; AMTS screening; and haemoglobin levels on admission.

### Nottingham Hip Fracture Score

In addition to the clinical information described, the NHFD Audit Programme also gave us an opportunity to include NHFS, which was agreed with Kent Surrey Sussex Academic Health Science Network for approximately 2 years of data collection, between 1/10/2015 and 7/12/2017. The NHFS has a maximum score of 10 points and is based on age (66–85 years = 3, ≥ 86 years = 4), sex (male = 1), admission haemoglobin (≤ 100 g/l = 1), AMTS (≤ 6 = 1), residence (living in an institution = 1), co-morbidities (≥ 2 = 1), active malignancy within 20 years (yes = 1)—these scores summate to a scale of between 0 and 10 [9].

### Categorisation of Variables

Mobilisation within 1 day after surgery was defined as patients with hip fractures who were able to start rehabilitation no later than the day after surgery [15]. Prolonged LOS was defined as a LOS > 17 days in hospital, *i.e.* in the upper quartile of LOS. Change in discharge destination was defined as those who came from their own home before hospital admission but did not return home directly after discharge and transferred to places where increased care was provided, including rehabilitation units, residential home or nursing care. Those who died in hospital were excluded from the analysis on discharge destination (*i.e.* only survivors to discharge were included).

### Statistical Analysis

Receiver operating characteristic (ROC) curves were constructed to determine the area under the curve (AUC) for the NHFS as a predictor of outcomes (in-patient mortality, prolonged LOS in hospital or discharge to residential/nursing care). Two-graph ROC curve analysis was conducted to optimise the selection of the maximum test accuracy for a given NHFS threshold value for identifying at-risk individuals—by plotting an overlapping graph of sensitivity and specificity curves as a function of the NHFS scores. The threshold *d*_0_ was obtained by interpolating from the intersection where sensitivity equals specificity (*θ*_0_), and the intermediate range (IR_90%_) was determined by the distance between the two points where sensitivity (lower limit) and specificity (upper limit) equal 90% [16–18]. These derived limits of IR_90%_ were used as cut-off values to define risk levels of NHFS for identification of outcomes; an NHFS below the lower limit of IR_90%_ indicates low risk, and above upper limit of IR_90%_ indicates high risk, while an NHFS within the IR_90%_ indicates intermediate risk.

Group data are given as mean values ± standard deviation (SD). Differences in LOS between NHFS risk groups were tested by ANOVA with *post hoc* analyses using a least-significant difference test where necessary. Differences between categorical outcome variables were assessed by Chi-squared tests. Logistic regression was conducted to assess the association of different NHFS risk groups with outcome measures. Analyses were performed using IBM SPSS Statistics, v23.0 (IBM Corp., Armonk, NY).

## Results

**Fig. 1:**
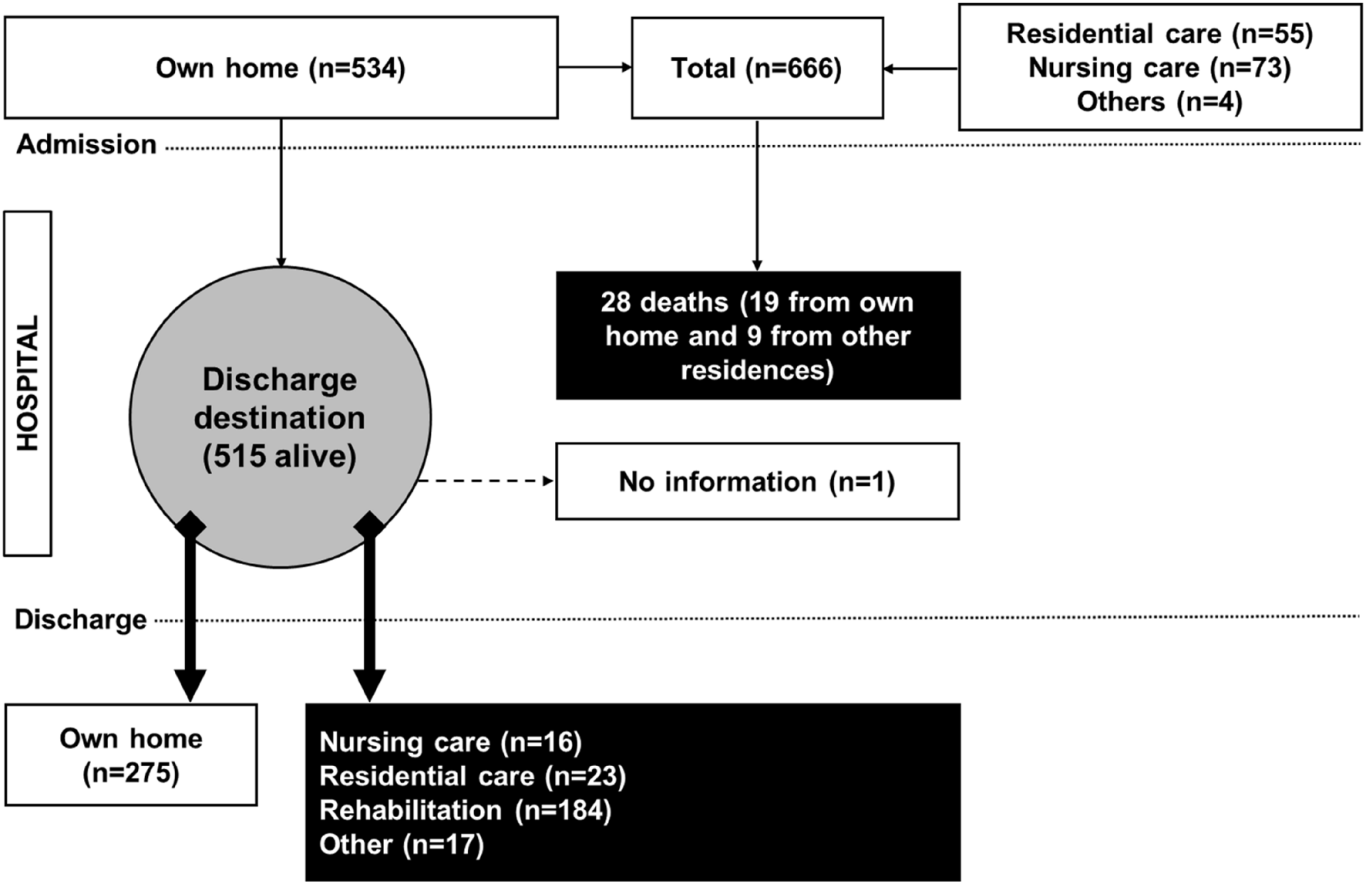
Flowchart showing patient distribution before and during hospital admission and on discharge

Data from a total of 666 patients (190 men) and (476 women) aged 60.2–103.4 years (mean = 84.1 years ± 8.4) were analysed for all outcomes, except for the analysis of discharge destination where patients had to be from their own home originally and survived to discharge (*n* = 515, see below). There were 534 patients (80.2%) who came from their own home, 55 (8.3%) came from residential care, and 73 (11.0%) from nursing care and 4 (0.6%) from other types of residence. Of those patients who came from their own home, 19 (3.6%) died in hospital and 515 survived for discharge, among whom 275 returned to their home, 184 transferred to rehabilitation, 23 went to residential care and 16 to nursing care and 17 to unknown destination (Fig. 1). Based on our data, the median LOS during admission was 11.2 (interquartile range = 7.3–17.1 days), which is slightly shorter than the value for the overall NHFD in England, Wales and Ireland (median LOS = 12 days, no interquartile range available) [12]. There were slightly higher proportions of left (51.4%) than right hip fractures (48.6%), in which 34.5% were intertrochanteric grade A1/A2, 3.0% intertrochanteric grade A3, 54.1% intracapsular displaced, 5.1% intracapsular undisplaced, and 3.3% subtrochanteric (Table 1).

**Table 1:** Characteristics of 666 patients admitted with hip fractures

	Proportion (%)
Sex distribution (women: men)	71.5: 28.5
Residence before admission	
Own home: residential care: nursing care	80.2: 11.0: 8.3
Fracture sides (left: right)	51.4: 48.6
Fracture type (IT-grade A1/A2: IT- grade A3: IC-displaced: IC-undisplaced: ST)	34.5: 3.0: 54.1: 5.1: 3.3
NHFS (0–3: 4: 5: 6: 7: 8: 9–10)	16.5: 28.7: 21.4: 16.5: 12.3: 4.1: 0.7
Mobile: failure to mobilise within 1 day after surgery	78.5: 21.5
LOS (< 17 days: ≥ 17)	75.2: 24.8
Discharge destination	
Own home/sheltered accommodation	53.4
Rehabilitation units	35.7
Residential or nursing home	7.6
Death	3.6

*IT* intertrochanteric, *IC* intracapsular, *ST* subtrochanteric

**Fig. 2:**
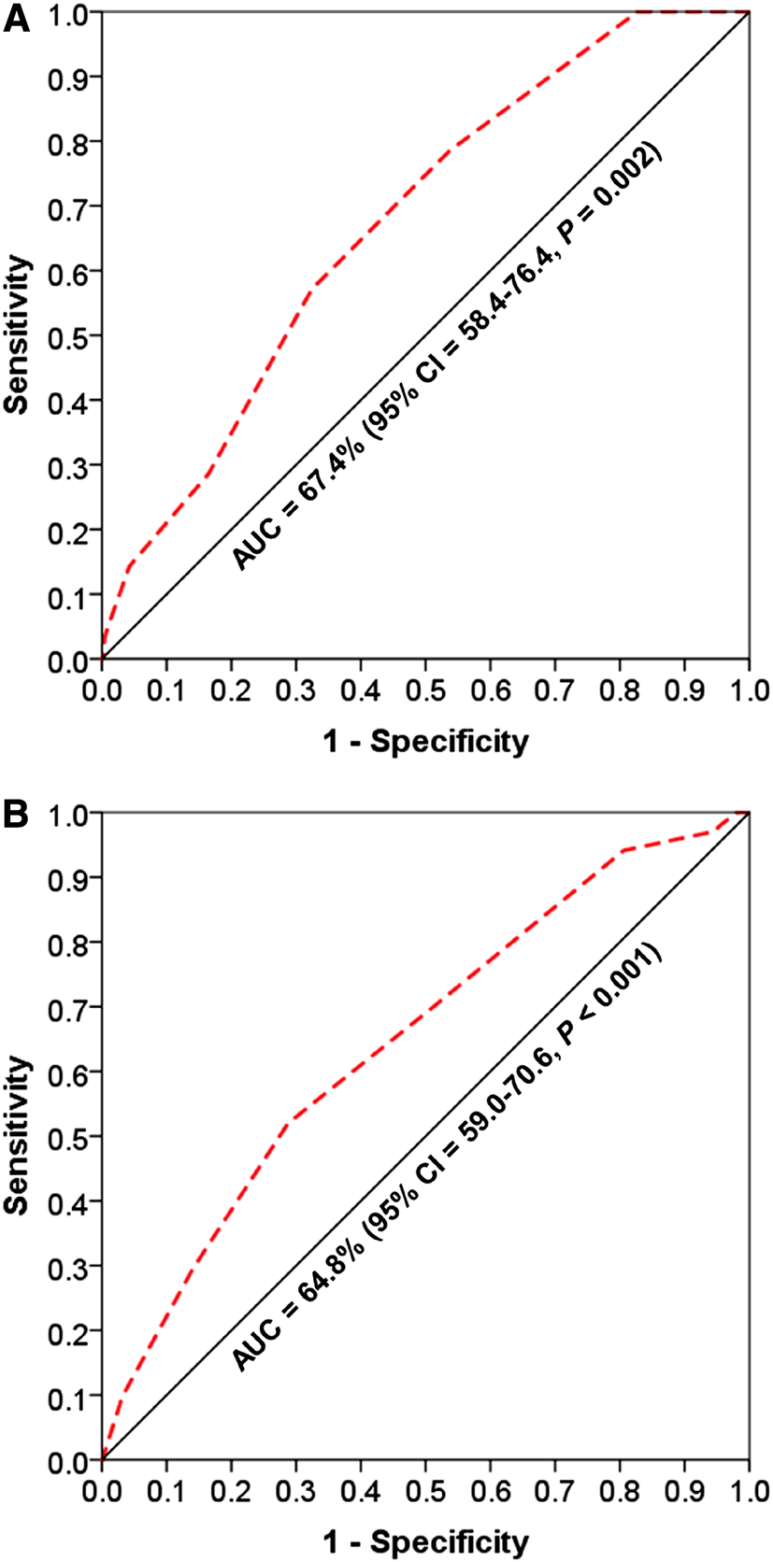
ROC curves (dotted lines) to estimate the ability of NHFS to predict in-patient mortality (**a**) and any two of the three outcomes (failure to mobilise within 1 day of surgery, prolonged LOS, and discharge to residential/nursing care) (**b**)

ROC analysis to generate AUC values showed that the NHFS as a predictor of failure to mobilise within 1 day of surgery was 56.0% (95% CI 50.7–61.3%, *P* = 0.027), prolonged LOS in hospital was 59.0% (95% CI 54.0–64.0%, *P* = 0.001), in-patient mortality was 67.4% (95% CI 58.4–76.4%, *P* = 0.002) (Fig. 2a) and discharge to residential/nursing care was 62.3% (95% CI 54.0–71.5%, *P* = 0.008). A composite variable, constructed from any two of the three outcomes (failure to mobilise within 1 day of surgery, prolonged LOS, and discharge to residential/nursing care) were shown to be more strongly associated with NHFS, with AUC rose to 64.8% (59.0–70.6%, *P* < 0.001) (Fig. 2b). The AUC increased further to 76.2% (95% CI 64.1–88.3%, *P* < 0.001) when NHFS was related to the composite of all of the three outcomes, but the event rate was too small (2.4%) for further reliable analysis.

**Fig. 3:**
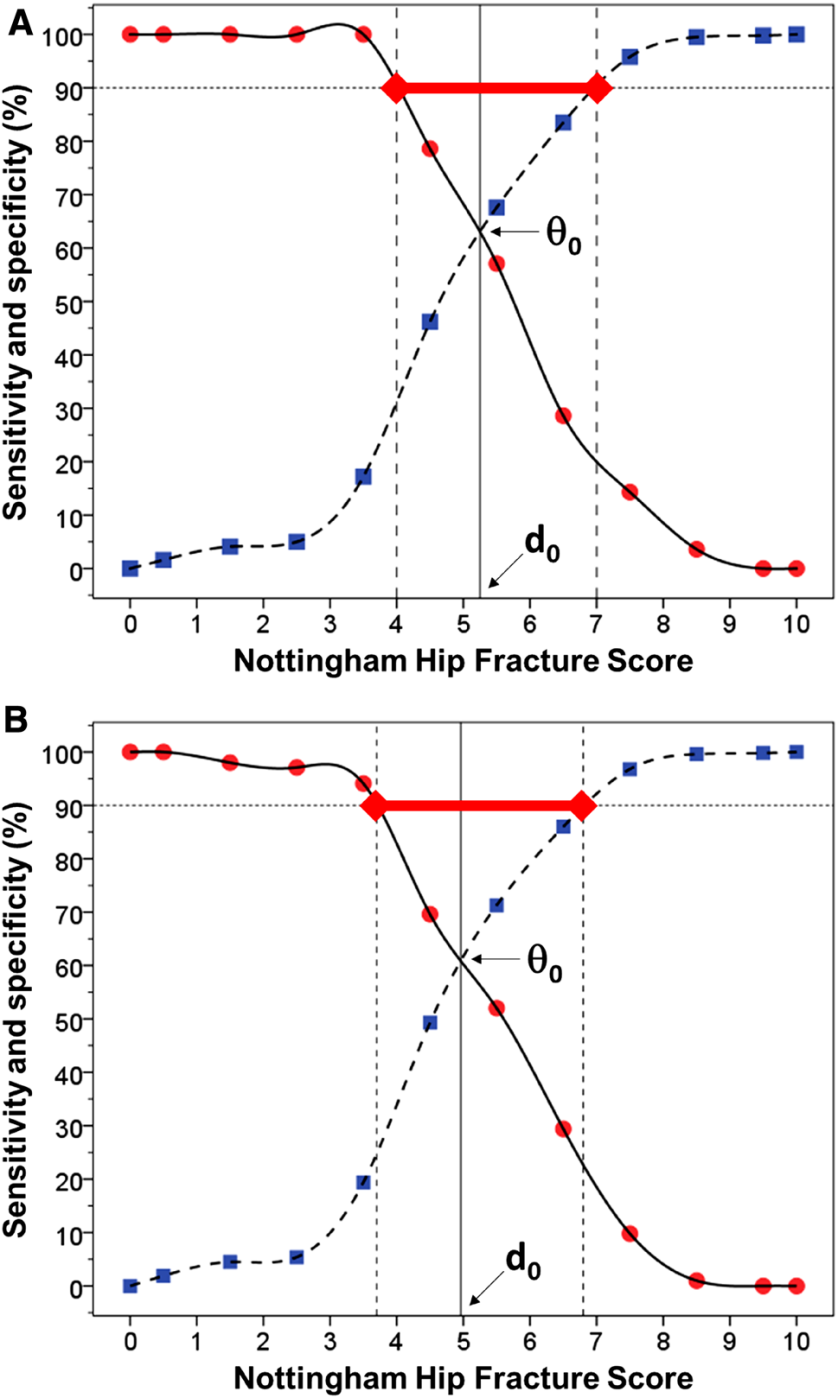
Two-graph ROC plot to identify in-patient mortality (**a**) and any two of the three outcomes (failure to mobilise within 1 day of surgery, prolonged LOS, of discharge to residential/nursing care) (**b**). These show the threshold of the NHFS index (*d*_0_) interpolated from the point where sensitivity (●) equals specificity ( ) (*θ*_0_), and the intermediate range (red bar) where sensitivity = 90% (lower limit) and specificity = 90% (upper limit)

Two-graph ROC plots showed the NHFS threshold (*d*_0_) where sensitivity equals specificity (*θ*_0_) for predicting in-patient mortality was 5.3 (IR_90%_ = 4.0–7.0—see Fig. 3a), failure to mobilise within 1 day of surgery was 4.8 (IR_90%_ = 3.6–7.0), prolonged LOS was 4.8 (IR_90%_ = 3.6–6.9), discharge to residential/nursing care was 4.8 (IR_90%_ = 3.7–6.4), and any two of the three outcomes (failure to mobilise, prolonged LOS or discharge to residential/nursing care) was 5.0 (IR_90%_ = 3.7–6.7)—see Fig. 3b); data are summarised in Table 2. Based on these results, NHFS thresholds at 4 and 7, corresponding to the lower and upper limits of IR_90%_, were selected for the categorisation of three risk groups for failure to mobilise within 1 day of surgery, prolonged LOS and mortality. In addition, similar thresholds at 4 and 6 were calculated for discharge to residential/nursing care. Thus, patients with an NHFS between 0 and 4 were considered to be in the low risk group, an NHFS of 5 + 6 (5 for residential/nursing care analysis) in an intermediate risk group, and between 7 and 10 (between 6 and 10 for residential/nursing care analysis) in high risk group.

**Table 2:** ROC and two-graph ROC analysis to assess the predictability of NHFS on outcomes and to determine cut-offs based on intermediate range where sensitivity and specificity equal 90% (IR_90%_)

	ROC analysis			Two-graph ROC analysis		
	AUC (%)	95% CI	*P*	Threshold (*d*_0_)^a^	Lower IR_90%_	Upper IR_90%_
Mortality	67.4	58.4–76.4	0.002	5.3	4.0 (4)	7.0 (7)
Failure to mobilise within 1 day of surgery	56.0	50.7–61.3	0.027	4.8	3.6 (4)	7.0 (7)
Prolonged LOS	59.0	54.0–64.0	0.001	4.8	3.6 (4)	6.9 (7)
Discharge to residential/nursing care	62.8	54.0–71.5	0.008	4.8	3.7 (4)	6.4 (6)
Any two of failure to mobilise, prolonged LOS or discharge to residential/nursing care	64.8	59.0–70.6	< 0.001	5.0	3.7 (4)	6.8 (7)
All three of failure to mobilise, prolonged LOS or discharge to residential/nursing care^b^	76.2	64.1–88.3	< 0.001	5.7	5.0 (5)	7.0 (7)

^a^Threshold at *d*_0_ indicates the intersection at which sensitivity and specificity are equal. Numbers in parentheses indicate threshold values used for subsequent analyses

^b^Event rate was too small (2.4%) for further analysis

Among the 666 patients studied, there were 301 patients (45.2%) in the low risk group, 142 (21.3%) in the intermediate risk group, and 223 (33.5%) in the high risk group. The corresponding values for the 515 patients in the discharge to residential/nursing care analysis were 264 (51.3%), 113 (21.9%), and 138 (26.8%), respectively.

There were significant differences between the three risk groups for failure to mobilise within 1 day of surgery: 18.9, 21.4, and 28.3% (*χ*^2^ = 4.3, *P* = 0.049), for prolonged LOS: 20.3, 26.3 and 33.9% (*χ*^2^ = 8.6, *P* = 0.015), for mortality: 2.0, 5.6 and 7.1% (*χ*^2^ = 7.1, *P* = 0.009), for discharge to residential or nursing care: 4.5, 8.8 and 12.3% (*χ*^2^ = 7.1, *P* = 0.017), and for and any two of the three outcomes (failure to mobilise, prolonged LOS or discharge to residential/nursing care): 10.5, 17.2 and 28.6% (*χ*^2^ = 19.2, *P* < 0.001), respectively. Compared with the low risk group, the high risk group had increased risk of failure to mobilise within 1 day of surgery by 1.7-fold (95% CI 1.0–2.8), prolonged LOS by 2.2-fold (95% CI 1.3–3.3), in-patient mortality by 3.8-fold (95% CI 1.5–9.9), discharge to residential/nursing care by 3.0 (95% CI 1.4–6.4), and any two of the three outcomes (failure to mobilise, prolonged LOS or discharge to residential/nursing care) by 3.4 (95% CI 1.9–6.0) (Table 3). Further adjustment for the sides and types of hip fracture did not alter the results.

**Table 3:** Rates and risk of mortality, prolonged LOS in hospital and discharge to residential/nursing care

	NHFS = 0–4 (*n* = 301)^a,b^	NHFS = 5 + 6 (*n* = 142)^b^				NHFS = 7–10 (*n* = 223)^b^			
	Event rates (%)	Event rates (%)	OR	95% CI	*P*	Event rates (%)	OR	95% CI	*P*
Inpatient mortality	2.0	5.6	2.19	0.69–6.85	0.187	7.1	3.80	1.46–9.88	0.006
Failure to mobilise within 1 day of surgery	18.9	21.4	1.17	0.77–1.77	0.466	28.3	1.69	1.03–2.79	0.040
Prolonged LOS in hospital	20.3	26.3	1.40	0.94–2.09	0.094	33.9	2.02	1.25–3.27	0.004
Discharge to residential/nursing care	4.5	8.8	2.04	0.85–4.87	0.109	12.3	2.95	1.37–6.37	0.006
Any two of failure to mobilise, prolonged LOS or discharge to residential or nursing care	10.5	17.2	1.77	1.07–2.93	0.025	28.6	3.40	1.94–5.99	< 0.001

^a^Reference group; ^b^For analysis of discharge to residential/nursing care, only those admitted from own home were selected: NHFS categories for this particular analysis were defined as 0–4 (*n* = 264), 5 (*n* 113) and 6–10 (*n* = 138)

**Fig. 4:**
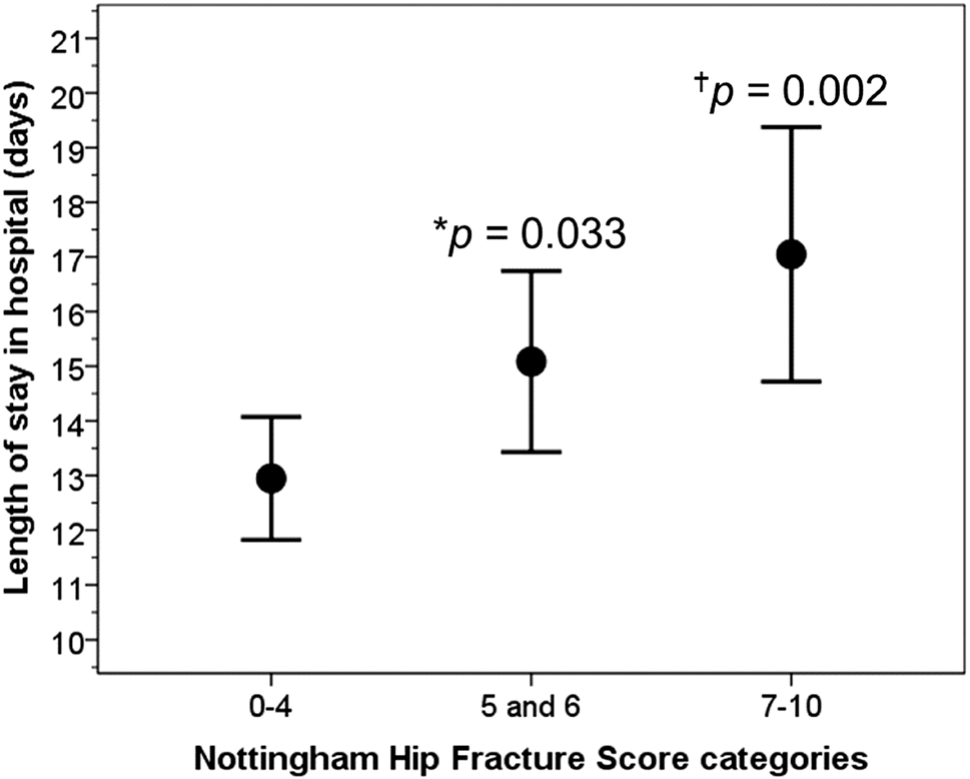
Length of stay in hospital according to NHFS risk groups

ANOVA showed significant differences in LOS in hospital between NHFS risk categories (*F* = 5.6, *P* = 0.004): 12.9, 15.1 and 17.0 days for NHFS 0–4, 5 + 6, and 7–10. Patients in the intermediate and in high risk group stayed in hospital longer by 2.1 days (95% CI 0.2 to 4.1) and 4.1 days (95% CI 1.5 to 6.7) than those in the low risk group (Fig. 4). There were no significant differences in LOS between intermediate and high risk groups.

## Discussion

This study found that a high NHFS (≥ 6 to identify risk of discharge to residential/ nursing care, or ≥ 7 to identify failure to mobilise within  1 day of surgery, prolonged LOS and mortality) was significantly associated with in-patient mortality by four-fold, failure to mobilise within 1 day of surgery and prolonged LOS in hospital by two-fold and discharge to residential/nursing care by three-fold. As far as we are aware, this is the first study to provide an evidence-based derivation of NHFS thresholds for the identification of patients at increased risk of adverse outcomes after a hip fracture. Our findings indicate that NHFS is applicable to identify a number of other clinical outcomes beyond its use for predicting post-discharge mortality. The three categories created from the two derived NHFS thresholds precisely provide different risk levels which are useful and practical in clinical settings for identifying at-risk patients.

Although the NHFS was originally developed to predict mortality [9, 10], for patients after a hip fracture, it has been shown here to relate to the proportion of those returning back to their own home, as well as their early discharge [11]. We have therefore further examined the ability of NHFS to predict a number of other outcome measures commonly experienced by patients admitted with a hip fracture. We observed that NHFS was associated with in-patient mortality with a predictive value (AUC) of 67.4%, which is modest but similar to figures reported from previous studies for predicting 30-day mortality (AUC range 67–72%) [10, 19, 20]. The ability of NHFS to predict other measures were also significant but weaker, especially for failure to mobilise within 1 day of surgery. However these findings, not been previously explored, provide further support to NHFS as a marker of adverse outcomes in patients with hip fracture. The strength of the association between NHFS and outcomes was further increased when individual outcomes (failure to mobilise within 1 day of surgery, prolonged LOS, and discharge to residential/nursing care) were combined using any two or all of the three outcomes—AUC rose to 64.8% and 76.2% respectively. Additional factors such as bone profile and renal function may provide further information on the prognosis and deserve further investigations, but this topic is beyond the scope of this study.

The association between NHFS and prolonged LOS in hospital observed in our study mirrors those reported by Moppett et al. [11] who showed increasing NHFS was related inversely to the proportions of patients being discharged early back to their own home. The extra days spent in hospital by patients with NHFS in the intermediate (2 days) or in the high risk (4 days) groups are similar to those observed in patients admitted with cognitive impairment or delirium [4]. Our observations of the association between high NHFS with discharge to residential/nursing care are also consistent with the findings of a negative correlation between NHFS and eventual return-to-home by Moppett et al. [11]. The threshold for identifying those at risk of being discharged to residential/nursing care was lower (6) than that for other outcomes (7) is likely due to differences in underlying health status between the two groups; patients from residential/nursing care before admission are likely to have poorer health than those from their own home.

The failure to mobilise within 1 day of surgery among those with high NHFS contributes to a delay in discharge or a need for increasing level of care. This notion is supported by our finding of an average LOS of 18.1 days for those who failed to mobilise within 1 day of surgery, compared with 13.5 days in those who could; a difference of 4.6 days (2.4–6.8 days, *P* < 0.001). There was also a higher proportion of those who were originally from their own home before admission being newly placed in a residential/nursing care (13.0% versus 6.3%, *P* = 0.024).

Previous studies have visually estimated various cut-offs for the NHFS (between 4 and 6) to predict 30-day mortality and selected a single threshold to separate low risk from high risk groups of patients, i.e. a binary variable [10, 21, 22]. In contrast, by employing a more precise two-graph ROC analysis to determine the upper and lower limits of IR_90%_, we were able to derive two thresholds in order to generate three categories of risk level (low, intermediate, and high risk groups).

It should be emphasised that the two-graph ROC analysis is employed to facilitate objective decisions on desired NHFS thresholds, which depends on a number of factors such as clinical benefits and risks, costs of interventions and physiological characteristics of the individual [23]. The risk of adverse outcomes follows stepwise increments with increasing levels of risk categories. If patients in the intermediate risk group were included, then the total numbers of at-risk patients would be larger but have a smaller proportional mortality rate, i.e. there would be more false negatives. Thus, the balance between resources and optimising recognition of risk should be taken into account when selecting an at-risk group.

The strengths of this study lie in its detailed information gathered in accordance with national guidelines [12, 13] in a wide range of age in older adults (60 to 103 years). The NHFS used in the present study has been well-validated and used widely in the UK and many countries. This study included dominantly white Caucasians representative of UK older individuals admitted with hip fractures [12]. Our performance including assessment, surgery and outcomes were mostly within or above expected range of national average [24]. There are other tools for assessing outcome measures with varying performance ability and practicality [25]. Caution should be taken when extrapolating our findings to other populations. We used the NHFS in this study because it was selected by the Royal College of Physicians for the NHFD Audit Programme. The majority of published data have focussed on medical-centric outcomes. Further studies are suggested to evaluate patient/carer-centric outcomes including shared decision making and self-management support, and patient participation in the planning and delivery of services.

In conclusion, a high NHFS associates with increased risk of in-patient mortality, prolonged LOS and discharge to residential/nursing care. This lends further support for the use of NHFS to identify other adverse events beyond its use in predicting post-discharge mortality.

## References

[1] Bliuc D, Alarkawi D, Nguyen TV, Eisman JA, Center JR (2015). Risk of subsequent fractures and mortality in elderly women and men with fragility fractures with and without osteoporotic bone density: the Dubbo Osteoporosis Epidemiology Study. J Bone Miner Res.

[2] Sullivan KJ, Husak LE, Altebarmakian M, Brox WT (2016). Demographic factors in hip fracture incidence and mortality rates in California, 2000–2011. J Orthop Surg Res.

[3] Papadimitriou N, Tsilidis KK, Orfanos P, Benetou V, Ntzani EE, Soerjomataram I, Künn-Nelen A, Pettersson-Kymmer U, Eriksson S, Brenner H, Schöttker B (2017). Burden of hip fracture using disability-adjusted life-years: a pooled analysis of prospective cohorts in the CHANCES consortium. Lancet Public Health.

[4] Lisk R, Uddin M, Parbhoo A, Yeong K, Fluck D, Sharma P, Lean ME, Han TS (2019). Predictive model of length of stay in hospital among older patients. Aging Clin Exp Res.

[5] Veronese N, Maggi S (2018). Epidemiology and social costs of hip fracture. Injury.

[6] https://www.ons.gov.uk/peoplepopulationandcommunity/birthsdeathsandmarriages/ageing/articles/livinglongerhowourpopulationischangingandwhyitmatters/2018-08-13. Accessed May 2020

[7] Basu N, Natour M, Mounasamy V, Kates SL (2016). Geriatric hip fracture management: keys to providing a successful program. Eur J Trauma Emerg Surg.

[8] Lisk R, Yeong K, Enwere P, Jenkinson J, Robin J, Irvin-Sellers M, Fluck D, Osmani A, Sharmin R, Sharma P, Fry CH, Han TS (2020). Ability of 4AT and its components to predict prolonged length of stay and mortality in hospital among patients admitted with hip fractures. Age Ageing.

[9] Maxwell M, Moran CG, Moppett IK (2008). Development and validation of a preoperative scoring system to predict 30 day mortality in patients undergoing hip fracture surgery. Br J Anaesth.

[10] Wiles MD, Moran CG, Sahota O, Moppett IK (2011). Nottingham Hip Fracture Score as a predictor of one year mortality in patients undergoing surgical repair of fractured neck of femur. Br J Anaesth.

[11] Moppett IK, Wiles MD, Moran CG, Sahota O (2012). The Nottingham Hip Fracture Score as a predictor of early discharge following fractured neck of femur. Age Ageing.

[12] Royal College of Physicians (2017) National Hip Fracture Database annual report 2017. RCP, London. https://www.nhfd.co.uk/. Accessed 1 Feb 2019

[13] National Hip Fracture Database National report 2013. Prepared on behalf of the Clinical Effectiveness and Evaluation Unit at the Royal College of Physicians. https://www.nhfd.co.uk/20/hipfractureR.nsf/0/CA920122A244F2ED802579C900553993/$file/NHFD%2520Report%25202013.pdf. Accessed 1 Feb 2019

[14] World Health Organization (2004). ICD-10: international statistical classification of diseases and related health problems: tenth revision.

[15] NICE quality standard QS16 (updated in 2016)

[16] Greiner M, Sohr D, Göbel P (1995). A modified ROC analysis for the selection of cut-off values and the definition of intermediate results of serodiagnostic tests. J Immunol Methods.

[17] Han TS, Van Leer EM, Seidell JC, Lean ME (1996). Waist circumference as a screening tool for cardiovascular risk factors: evaluation of receiver operating characteristics (ROC). Obes Res.

[18] Han TS, Gulli G, Affley B, Fluck D, Fry CH, Barrett C, Kakar P, Sharma S, Sharma P (2019). New evidence-based A1, A2, A3 alarm time zones for transferring thrombolysed patients to hyper-acute stroke units: faster is better. Neurol Sci.

[19] Nijmeijer WS, Folbert EC, Vermeer M, Slaets JP, Hegeman JH (2016). Prediction of early mortality following hip fracture surgery in frail elderly: the Almelo Hip Fracture Score (AHFS). Injury.

[20] Jonsson MH, Bentzer P, Turkiewicz A, Hommel A (2018). Accuracy of the physiological and operative severity score for the enUmeration of mortality and morbidity score and the Nottingham risk score in hip fracture patients in Sweden—a prospective observational study. Acta Anaesthesiol Scand.

[21] Moppett IK, Parker M, Griffiths R, Bowers T, White SM, Moran CG (2012). Nottingham Hip Fracture Score: longitudinal and multi-centre assessment. Br J Anaesth.

[22] Rushton PR, Reed MR, Pratt RK (2015). Independent validation of the Nottingham Hip Fracture Score and identification of regional variation in patient risk within England. Bone Joint J.

[23] Zou KH, O’Malley AJ, Mauri L (2007). Receiver-operating characteristic analysis for evaluating diagnostic tests and predictive models. Circulation.

[24] https://www.nhfd.co.uk/20/nhfdcharts.nsf/fmbenchmarks?ReadForm=&report=outcomes&readform=&year=2017. Accessed 1 Feb 2019

[25] Marufu TC, Elphick HL, Ahmed FB, Moppett IK (2019). Short-term morbidity factors associated with length of hospital stay (LOS): development and validation of a Hip Fracture specific postoperative morbidity survey (HF-POMS). Injury.

